# Therapeutic Potential of Mesenchymal Stem Cells and Their Secretome in the Treatment of SARS-CoV-2-Induced Acute Respiratory Distress Syndrome

**DOI:** 10.1155/2020/1939768

**Published:** 2020-11-20

**Authors:** Carl Randall Harrell, Biljana Popovska Jovicic, Valentin Djonov, Vladislav Volarevic

**Affiliations:** ^1^Regenerative Processing Plant, LLC, 34176 US Highway 19 N Palm Harbor, Palm Harbor, Florida, USA; ^2^Department of Infectious Diseases, Faculty of Medical Sciences, University of Kragujevac, Svetozara Markovica 69, 34000 Kragujevac, Serbia; ^3^Institute of Anatomy, University of Bern, 2 Baltzerstrasse, 3012 Bern, Switzerland; ^4^Department for Microbiology and Immunology, Center for Molecular Medicine and Stem Cell Research, Faculty of Medical Sciences, University of Kragujevac, 69 Svetozar Markovic Street, Kragujevac, Serbia

## Abstract

Severe acute respiratory syndrome coronavirus 2 (SARS-CoV-2), the etiological agent responsible for the development of a new coronavirus disease (COVID-19), is a highly transmittable virus which, in just ten months, infected more than 40 million people in 214 countries worldwide. After inhalation, aerosols containing SARS-CoV-2 penetrate to the depths of the lungs and cause severe pneumonia, alveolar injury, and life-threatening acute respiratory distress syndrome (ARDS). Since there are no specific drugs or vaccines available to cure or prevent COVID-19 infection and COVID-19-related ARDS, a new therapeutic agent which will support oxygen supply and, at the same time, efficiently alleviate SARS-CoV-2-induced lung inflammation is urgently needed. Due to their potent immuno- and angiomodulatory characteristics, mesenchymal stem cells (MSCs) may increase oxygen supply in the lungs and may efficiently alleviate ongoing lung inflammation, including SARS-CoV-2-induced ARDS. In this review article, we described molecular mechanisms that are responsible for MSC-based modulation of immune cells which play a pathogenic role in the development of SARS-CoV-2-induced ARDS and we provided a brief outline of already conducted and ongoing clinical studies that increase our understanding about the therapeutic potential of MSCs and their secretome in the therapy of COVID-19-related ARDS.

## 1. Introduction

From December 2019, the world population has been faced with a new coronavirus disease (COVID-19) caused by the novel severe acute respiratory syndrome coronavirus (SARS-CoV-2) [1]. As a highly transmittable virus, SARS-CoV-2 rapidly spread and, in just ten months, infected more than 40 million people in 214 countries worldwide [2].

**Figure 1:**
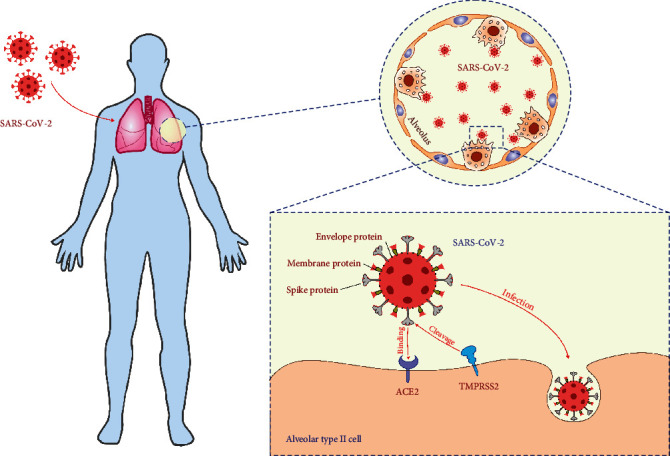
Cellular entry of SARS-CoV-2. Envelope, membrane, and spike proteins form the SARS-CoV-2 protein interface to the external environment. After inhalation, aerosols containing SARS-CoV-2 penetrate the depths of the lungs and cause severe pneumonia, alveolar injury, and acute respiratory distress syndrome (ARDS). The initial step in the development of SARS-CoV-2-induced ARDS is the interactions between the SARS-CoV-2 spike protein and angiotensin-converting enzyme 2 (ACE2) and transmembrane protease serine 2 (TMPRSS2) of alveolar type II cells. TMPRSS2 splits the spike protein into two functional subunits, S1 which interacts with ACE2 and S2 that is further cleaved and activated by TMPRSS2. This structural and conformational change in the spike protein facilitates fusion of viral envelope with the cell membrane of AT2 cells, enabling endocytic entry of the virus in the target cell.

Since the primary route of transmission of SARS-CoV-2 is through respiratory droplets, the lungs are severely injured in COVID-19 patients [3]. After inhalation, aerosols containing SARS-CoV-2 penetrate the depths of the lungs and cause severe pneumonia, alveolar injury, and life-threatening acute respiratory distress syndrome (ARDS) [4]. The initial step in the development of SARS-CoV-2-induced ARDS is the interactions between the SARS-CoV-2 spike (S) protein and angiotensin-converting enzyme 2 (ACE2) and transmembrane protease serine 2 (TMPRSS2) of alveolar type II cells (AT2) (Figure 1) [3]. TMPRSS2 splits the S protein into two functional subunits, S1 which interacts with ACE2 and S2 that is further cleaved and activated by TMPRSS2. This structural and conformational change in the S protein facilitates fusion of viral envelope with the cell membrane of AT2 cells, enabling endocytic entry of the virus in the target cell. Immediately after viral RNA is released into the cytoplasm of AT2 cell, the translational machinery of AT2 cell is used for the synthesis of the essential structural and functional viral proteins (proteases 3CLpro and PLpro, RNA-dependent RNA polymerase and helicase) [3, 4]. These proteins, as well as other structural components of SARS-CoV-2, are recognized by immune cells of innate (macrophages, dendritic cells (DCs), natural killer (NK), and natural killer T (NKT) cells) and acquired immunity (T and B lymphocytes) [5]. In the majority of patients, activated immune cells efficiently eliminate the virus and the disease is either asymptomatic or manifested by fever, cough, and mild lung inflammation [6]. However, in some patients, SARS-CoV-2 overactivates immune cells, eliciting strong immune response in the lungs. Excessive production and release of inflammatory cytokines and chemokines (interleukin- (IL-) 2, IL-6, IL-7, tumor necrosis factor-alpha (TNF-*α*), granulocyte colony-stimulating factor (G-CSF), monocyte chemoattractant protein-1 (MCP-1), CXCL10, and CCL3) result in the severe cytokine storm, followed by the massive influx of circulating granulocytes and monocytes in the inflamed lungs, which subsequently leads to the development of lung edema, dysfunction of the air exchange, and ARDS [4–6].

Currently, no specific drugs or vaccines are available to cure or prevent COVID-19 infection [7–4]. Several drugs, known to act on different stages of both the infection and host response, including Camostat and Nafamostat (which negatively influence S protein: ACE2/TMPRSS2 interaction), Remdesivir (that interferes with viral replication), Sarilumab and Tocilizumab (which inhibit IL-6-driven inflammation), and Dexamethasone (that prevents SARS-CoV-2-induced cytokine storm), managed to improve the clinical symptoms in some SARS-CoV-2-infected patients (Table 1) [7–14]. However, none of these drugs were able to prevent or attenuate the cytokine storm in the lungs, and therefore, patients suffering from SARS-CoV-2-induced ARDS usually need oxygen supply or mechanical ventilation in addition to antiviral therapy [4, 8]. Accordingly, a new therapeutic agent which will support oxygen supply and, at the same time, efficiently alleviate the SARS-CoV-2-induced cytokine storm is urgently needed.

Due to their potent immuno- and angiomodulatory characteristics, mesenchymal stem cells (MSCs) have been used in a large number of experimental and clinical trials as new therapeutic agents for the treatment of inflammatory and degenerative diseases [15]. MSCs may be easily sourced from various tissues (bone marrow (BM), blood, umbilical cord (UC), Wharton's jelly (WJ), adipose tissue, dental pulp (DP), synovium, amniotic fluid, etc.) and expanded *in vitro* to achieve a sufficient cell number for clinical application [16]. Due to the reduced expression of major histocompatibility class (MHC) II antigens, MSCs are considered as hypoimmunogenic cells which usually evade allogeneic rejection after engraftment in MHC-mismatched recipients [17]. Therefore, MSCs may efficiently suppress detrimental immune responses and alleviate ongoing lung inflammation including SARS-CoV-2-induced ARDS [18, 19]. In this review article, we described molecular mechanisms that are responsible for MSC-based modulation of immune cells which play a pathogenic role in the development of SARS-CoV-2-induced ARDS and we provided a brief outline of already conducted and ongoing clinical studies that increase our understanding about the therapeutic potential of MSCs and their secretome in the therapy of COVID-19-related ARDS. An extensive literature review was carried out in May 2020 across several databases (MEDLINE, EMBASE, Google Scholar, and ClinicalTrials.gov). Keywords used in the selection were as follows: “mesenchymal stem cells (MSCs)”, “secretome”, “exosomes (Exos)”, “immunomodulation”, “SARS-CoV-2”, “COVID-19”, and “ARDS”. All journals were considered, and an initial search retrieved 32 articles. The abstracts of all these articles were subsequently reviewed by two of the authors (CRH and VV) to check their relevance to the subject of this manuscript. Eligible studies had to delineate therapeutic effects of MSCs in SARS-CoV-2-induced ARDS, and their findings were analyzed in this review.

## 2. Molecular and Cellular Mechanisms Responsible for MSC-Based Attenuation of ARDS

ARDS is a severe clinical syndrome which develops as a result of an acute, detrimental systemic inflammatory response in the lungs [20, 21]. Lung DCs, activated by inhaled pathogens, increase the expression of costimulatory and MHC molecules which migrate to the regional lymph nodes to present pathogen-associated molecular patterns (PAMPs) to the naive T cells [20]. DC-derived IL-12 induces generation of CXCR3-expressing, TNF-*α*- and IFN-*γ*-producing Th1 cells, while DC-sourced IL-23, IL-1*β*, IL-6, and TGF-*β* are responsible for the development of IL-17-producing Th17 cells. Inflammatory Th1 and Th17 cells activate alveolar macrophages and lung-infiltrated neutrophils to produce inflammatory mediators (nitric oxide (NO), IL-6, IL-8, TNF-*α*, and IL-1*β*) that cause injury and dysfunction of endothelial cells increasing vascular permeability [20]. Additionally, activated lung DCs and alveolar macrophages secrete large amount of neutrophil-, monocyte-, and lymphocyte-attracting chemokines (IL-8, CCL2, monocyte chemoattractant protein-1 (MCP-1), and CXCR3), resulting in the increased influx of circulating leucocytes in the inflamed lungs [21]. Activated neutrophils, macrophages, and T cells release proteolytic enzymes, reactive oxygen species (ROS), and other potent cytotoxic mediators which induce injury of AT2 and endothelial cells [21]. Inflammation-induced vascular permeability causes exudation from the plasma to the alveolar spaces which decreases alveolar fluid clearance and leads to the development of lung edema. The disruption of the alveolar-epithelial barrier, accompanied with interstitial edema and infiltration of inflammatory cells, leads to the acute respiratory failure [18, 20].

**Figure 2:**
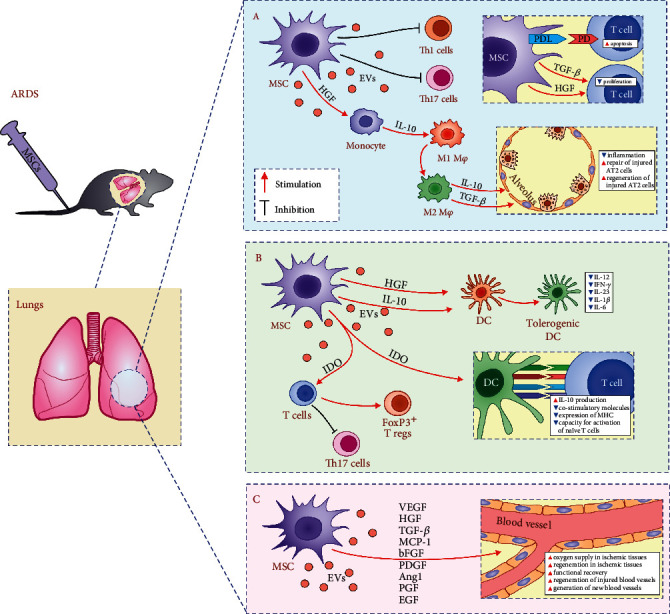
Molecular mechanisms responsible for MSC-based attenuation of ARDS. MSCs, in juxtacrine (cell-to cell-contact dependent) and paracrine manner (through the production of immunomodulatory factors), alleviate lung inflammation and ARDS by suppressing proliferation and effector functions of inflammatory M1 macrophages and Th1 and Th17 cells (a) and by inducing generation of tolerogenic DCs and expansion of immunosuppressive Tregs (b). Through the production of angiomodulatory factors, MSCs improve oxygen supply enabling enhanced regeneration and functional recovery from ARDS.

A large number of experimental studies revealed that MSCs in juxtacrine (cell-to-cell contact-dependent) and/or paracrine manner (through the production of trophic and immunoregulatory factors) efficiently suppress ARDS by modulating phenotype and function of Th1, Th17 cells, DCs, and alveolar macrophages in the inflamed lungs (Figure 2) [18, 22]. MSCs prevent proliferation and expansion of inflammatory, IFN-*γ*- and IL-17-producing CD4+ Th1 and Th17 cells [23]. MSCs express program death (PD) ligand and induce PD receptor-dependent apoptosis of effector T cells, alleviating their number in injured lungs [18]. Additionally, MSCs produce transforming growth factor-beta (TGF-*β*) which suppresses activation of the Jak-Stat signaling pathway in activated T cells, causing the G1 cell cycle arrest [24]. MSC-sourced HGF acts synergistically with TGF-*β* in the MSC-mediated suppression of T cell proliferation [24]. MSC-derived HGF induces enhanced production of immunosuppressive IL-10 in monocytes enabling conversion of inflammatory M1 macrophages in alternatively activated M2 phenotype [24]. M2 macrophages, in turn, produce anti-inflammatory IL-10 and TGF-*β* that alleviate ongoing inflammation and promote repair and regeneration of injured AT2 cells [18].

MSC-sourced prostaglandin E2 (PGE2) and IL-10 induce generation of tolerogenic phenotype in DCs alleviating their antigen-presenting properties [24]. MSC-primed tolerogenic DCs have reduced capacity for the production of Th1- and Th17-related cytokines (IL-12, IFN-*γ*, IL-23, IL-1*β*, and IL-6) [18]. Accordingly, reduced presence of activated DCs and lower numbers of inflammatory Th1 and Th17 cells were observed in the lungs of MSC-treated animals [18]. MSC-derived indoleamine 2,3-dioxygenase (IDO) is crucially important for MSC-based modulation of DC : T cell cross-talk in inflamed lungs [18]. MSCs in IDO-dependent manner enhance production of immunosuppressive IL-10 in lung-infiltrated DCs and suppress expression of costimulatory and MHC molecules on their membranes, downregulating their capacity for activation of naive T cells [16, 24]. Additionally, MSC-derived IDO induces expansion of immunosuppressive, regulatory FoxP3-expressing T cells and prevents their transdifferentiation in inflammatory Th17 cells [16].

In addition to the suppression of detrimental immune response in the inflamed lungs, MSC-based therapy of ARDS is, at least partially, reliant on their potent angiomodulatory characteristics [18, 25]. A large number of experimental and clinical studies demonstrated that injection of MSCs or their secretome significantly improved oxygen supply in ischemic tissues enabling their enhanced regeneration and functional recovery [25]. MSC-dependent neovascularization is mainly reliant on the capacity to produce large number of proangiogenic factors (vascular endothelial growth factor (VEGF), HGF, TGF-*β*, MCP-1, basic fibroblast growth factor, platelet-derived growth factor, angiopoietin-1, placental growth factor, and epidermal growth factor) which support regeneration of injured and induce generation of new blood vessels [24, 25].

## 3. MSC-Based Therapy of ARDS: Evidence from Experimental Studies and Clinical Trials

Therapeutic potential of MSCs in the treatment of ARDS has been demonstrated in experimental and clinical studies [18]. MSCs enhanced regeneration of AT2 and endothelial cells, alleviated inflammation, reduced pulmonary edema, improved oxygenation, and prolonged survival of mice suffering from ARDS [18]. Beneficial effects of MSCs were due to the enhanced immunosuppression and improved oxygenation in the injured lungs [18]. MSC-based inhibition of detrimental immune response in the lungs mainly relied on the anti-inflammatory effects of MSC-sourced PGE2 and IL-10 [16, 24]. MSC-derived PGE2 induced generation of the immunosuppressive M2 phenotype in alveolar macrophages and increased their capacity for IL-10 production [26]. IL-10, released by MSCs and M2 macrophages, attenuated secretion of inflammatory TNF-*α* in lung-infiltrated lymphocytes and inhibited production of ROS and other inflammatory mediators in neutrophils and monocytes [26]. MSC-derived proresolving mediator lipoxin A4 was mainly responsible for reduced pulmonary edema [27], while MSC-sourced VEGF and HGF improved vascularization and oxygenation in injured lungs by promoting generation of new blood vessels and by preventing apoptosis of injured endothelial cells [28, 29].

In a similar manner as it was observed in mice, intravenous injection of MSCs was well tolerated in patients who suffered from moderate and severe ARDS [30, 31]. As noticed in a clinical trial conducted by Wilson and colleagues (NCT01775774), adverse events, clinical instability, or dose-limiting toxicity was not observed in any of the nine patients that received allogeneic BM-MSCs (1, 5, or 10 million cells/kg) [30]. Based on these promising results, Matthay and colleagues conducted a larger randomized, multicenter, phase 2 clinical study in five university medical centers in the United States (NCT02097641). The trial enrolled 60 adult ARDS patients who intravenously received either a single dose of allogeneic BM-MSCs (10 million cells/kg; 40 patients) or placebo (Plasma-Lyte A; 20 patients) [31]. One of the MSC-treated patients had a fatal cardiopulmonary arrest 20 hours after MSC injection, but the authors explained that this patient had a preexisting history of coronary artery disease and concluded that his death was not a consequence of MSC infusion [31]. Accordingly, as the most important finding of their study, Matthay and colleagues highlighted that intravenous administration of MSCs was a safe therapeutic approach for the treatment of patients with moderate and severe ARDS [31]. Although clinical parameters did not differ significantly between MSC and placebo-treated patients, post hoc analyses showed a trend for improvement in the oxygenation index in MSC-treated patients [31]. However, it should be noted that Matthay and colleagues indicated that this analysis was limited because of some missing data and that MSC-induced improvement in oxygenation index should not be overinterpreted as a favorable finding until it will be assessed further in a larger clinical trial [31]. Intriguingly, mortality at 28 and 60 days was not significantly higher in the MSC group than in the placebo group. The number of ventilator-free and organ-failure-free days was not significantly lower in the MSC group while the number of intensive-care-free days was higher in the placebo group, suggesting that MSC-based therapy was less effective than placebo [31]. Matthay and colleagues explained that these differences were most probably a consequence of the fact that, according to the baseline respiratory variables (minute ventilation, respiratory rate, and oxygenation index), patients who were selected for MSC treatment had more severe respiratory failure than patients who were assigned to the placebo group [31]. Despite this reasonable explanation, the high disease severity score noticed in MSC-treated patients raises serious concerns about the therapeutic efficacy of MSCs in ARDS treatment and should be evaluated in detail in upcoming larger clinical trials.

## 4. Therapeutic Potential of MSCs in the Treatment of SARS-CoV-2-Induced ARDS

**Figure 3:**
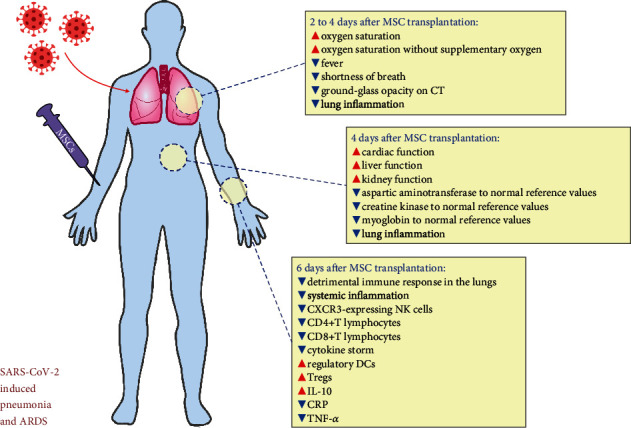
MSC-based effects observed in a patient with critically severe COVID-19 infection. MSCs significantly improved oxygenation, suppressed detrimental immune response in the lungs, alleviated systemic inflammation, and attenuated SARS-CoV-2 induced ARDS in a patient with critically severe COVID-19 infection.

Beneficial effects of MSCs in the treatment of SARS-CoV-2-induced pneumonia and ARDS have been demonstrated in a clinical study conducted by Leng and coworkers (Figure 3) [32]. They demonstrated that systemic, intravenous administration of MSCs (1 × 10^6^ cells/kg) efficiently alleviated lung inflammation and significantly improved pulmonary function in patients suffering from SARS-CoV-2-induced pneumonia [32]. Ten confirmed COVID-19 patients were enrolled in this study. MSCs were transplanted in 1 patient with critically severe COVID-19 and 4 patients with severe and 2 patients with mild disease, while 3 patients with severe COVID-19, who received placebo, were assigned to the control group. According to the protocol, MSCs were injected when COVID-19-related symptoms and/or signs were getting worse, despite the use of standard medicaments [32]. Infusion-related reactions, allergies, secondary infections, and life-threatening adverse events were not observed in MSC-treated patients during the 14 days of follow-up, indicating that systemic administration of MSCs was a safe therapeutic approach for the treatment of COVID-19 patients. Evaluation of COVID-19-related symptoms and signs (fever (38.5°C ± 0.5°C), shortness of breath, cough, sore throat, and weakness) and the analysis of clinical, laboratory, and radiological outcomes (plasma levels of C-reactive protein (CRP) and cytokines, total number of lymphocytes and their subpopulations in the blood, the oxygen saturation, respiratory rate, and chest computed tomography (CT)) revealed that MSCs efficiently attenuate SARS-CoV-2-induced pneumonia and ARDS [32]. About 2 to 4 days after MSC transplantation, all COVID-19-related symptoms disappeared in all patients, and the oxygen saturations rose to ≥95% at rest, with or without oxygen uptake (5 liters per minute). Importantly, the oxygen saturation, without supplementary oxygen, rose from 89% to 98% in MSC-treated patients with critically severe disease, suggesting that pulmonary alveoli completely regained their air-exchange function. Both fever and shortness of breath disappeared on the 4th day after MSC transplantation. CT findings confirmed beneficial effects of MSCs. Ground-glass opacity, initially observed in the lungs of critically severe COVID-19 patients, almost completely disappeared as a result of MSC-based therapy. Two weeks after MSC infusion, only little ground-glass opacity was locally noticed in the lungs of MSC-treated patients, confirming MSC-dependent attenuation of lung inflammation. Additionally, 4 days after MSC administration, the levels of aspartic aminotransferase, creatine kinase, and myoglobin decreased to normal reference values, indicating that MSCs improved cardiac, liver, and kidney functions in COVID-19 patients, preventing the development of SARS-CoV-2-induced multiple organ dysfunction syndrome [32].

Importantly, MSCs suppressed detrimental immune responses in the lungs and alleviated systemic inflammation in COVID-19 patients [32]. The mass cytometry results of peripheral blood mononuclear cells revealed that MSCs, 6 days after transplantation, completely altered the phenotype of immune cells in SARS-CoV-2-infected patients. Significant decrease of inflammatory, overactivated CXCR3-expressing NK cells and CD4+ and CD8+ T lymphocytes (which cause the cytokine storm in SARS-CoV-2 injured lungs) and significant increase in immunosuppressive, regulatory DCs and Tregs (which promote repair and regeneration of inflamed lungs) were observed in the blood of MSC-treated patients with critically severe disease [32]. In line with these findings, remarkably increased anti-inflammatory IL-10 and significantly decreased CRP and TNF-*α* were observed in plasma samples of MSC-treated patients, confirming MSC-based suppression of detrimental immune response in the lungs and alleviation of systemic inflammation in COVID-19 patients [32].

RNA-seq analysis of transplanted MSCs revealed that potent immunosuppressive and proangiogenic properties of MSCs were responsible for their therapeutic effects in COVID-19 treatment [32]. MSC-based improvement in oxygen supply could be a consequence of increased expression of angiomodulatory factors VEGF, EGF, and FGF, while enhanced expression of anti-inflammatory TGF-*β* and HGF could be responsible for MSC-dependent immunosuppression. Importantly, RNA-seq analysis revealed that MSCs did not express ACE2 and TMPRSS2 [32]. Therefore, it is highly expected that MSCs could not be infected by SARS-CoV-2.

## 5. MSC-Based Therapy of SARS-CoV-2-Induced Lung Injury: Ongoing Clinical Trials

Promising results obtained by Leng and colleagues [32] encouraged many research groups to conduct clinical trials which are going to further explore and confirm therapeutic potential of MSCs in the treatment of SARS-CoV-2-induced pneumonia and ARDS.

Wang and coworkers are currently recruiting 90 COVID-19 patients for participation in a prospective, double-blind, multicenter, randomized clinical trial which will evaluate therapeutic effects of MSCs in the treatment of severe SARS-CoV-2-induced pneumonia (NCT04288102). The experimental group will consist of 60 patients who will, in addition to conventional treatment, intravenously receive 3 doses of MSCs (4 × 10^7^ cells per dose, injected on days 0, 3, and 6), while the control group will consist of 30 patients who are going to receive combination of conventional treatment and placebo. Beneficial effects of MSCs in alleviation of lung injury will be evaluated by chest CT, Modified Medical Research Council dyspnea scale, 6-minute walk test, maximum vital capacity, diffusing capacity, oxygen saturation, and oxygenation index, while MSC-based immunosuppression will be examined by the immunophenotyping of peripheral blood mononuclear cell analysis of COVID-19 patients during the 90 days of follow-up.

Karaoz and colleagues recently elicited a clinical trial in which patients suffering from SARS-CoV-2-induced lung injury will either receive standard antiviral therapy or MSCs (NCT04392778). MSCs (3 × 10^6^/infusion) will be intravenously injected in 10 COVID-19 patients in 3 doses, at days 1, 3, and 6. The estimated study completion date is September 2020.

A large randomized, double-blind, placebo-controlled clinical trial designed by Gelijns and coworkers (NCT04371393) is currently recruiting patients. According to the study protocol, 300 patients suffering from SARS-CoV-2-induced ARDS will be assigned to either the experimental group (patients will intravenously receive MSCs (remestemcel-L) plus standard of care) or the control group (will receive placebo (Plasma-Lyte) plus standard of care). MSCs (2 × 10^6^kg) or placebo will be administered twice during the first week of treatment, specifically with the second infusion at 4 days following the first infusion. Changes from baseline in (a) severity of ARDS according to Berlin Criteria, (b) Clinical Improvement Scale, and (c) serum levels of inflammatory markers (CRP, IL-6, IL-8, and TNF-*α*) will be used for the evaluation of MSC-based therapeutic effects. Patients will be followed for 90 days, while pulmonary symptoms will be assessed at 6 and 12 months. The estimation study completion date is April 2022.

González-Vallinas and colleagues will be conducting a double-blind, placebo-controlled, randomized clinical trial in which 24 patients with severe COVID-19 pneumonia will receive either single dose of MSCs (1 million cells/kg) or placebo by intravenous injection (NCT04361942). In addition to the analysis of MSC-based therapeutic effects on lung function, results of this study should further emphasize the importance of MSC-dependent immunomodulation for the beneficial effects of MSCs in alleviation of SARS-CoV-2-induced lung injury and inflammation. For this purpose, González-Vallinas and coworkers will analyze whether MSCs altered total number and phenotype of circulating NK cells and T and B lymphocytes and will determine serum levels of inflammatory (IL-6, TNF-alpha) and immunosuppressive (IL-10) cytokines. The study is currently recruiting patients, and the estimated completion date is December 2020.

In the clinical trial which is currently recruiting patients (NCT042521188), Wang and colleagues will analyze whether MSCs could enhance therapeutic effects of conventional antiviral drugs in the treatment of SARS-CoV-induced pneumonia. From 20 recruited patients, 10 patients will receive conventional treatment while the other 10 patients will receive combination of conventional treatment and MSCs (3 × 10^7^ cells), which will be intravenously injected 3 times (at days 0, 3, and 6). Improvement of clinical symptoms (duration of fever, restoration of respiratory, liver, and kidney function), phenotype of circulating T cells, and 28-day mortality of MSC-treated COVID-19 patients will be monitored for 180 days. The estimated study completion date is December 2021.

A similarly designed study, which will evaluate the beneficial effects of DP-MSCs in the treatment of severe pneumonia of COVID-19 patients, is currently recruiting patients (NCT04336254). Twenty patients suffering from severe SARS-CoV-2-induced pneumonia (manifested by respiratory distress, respiratoryrate > 30 times/min; resting oxygen saturation of 93% or less; arterial partial pressure of oxygen/oxygen concentration 300 mmHg) will be randomly divided in two groups to receive either conventional treatment or combination of conventional therapy and DP-MSCs (3 × 10^7^ cells) which will be intravenously injected on days 1, 4, and 7. DP-MSC-mediated alleviation of lung inflammation will be confirmed by CT while total number of CD16+/CD56+ NK cells, CD3+/CD4+/CD8+ T lymphocytes, and CD19+ B cells in the blood; titer of IgA, IgG, IgM, and IgE; and concentration of Th1-related (IL-1*β*, IL-2, TNF-*α*, and IFN-*γ*) and Th2-related cytokines (IL-4, IL-6, and IL-10) will be determined to evaluate MSC-dependent suppression of detrimental immune response. The estimated study completion date is March 2021.

Therapeutic potential of WJ-MSCs in the treatment of COVID-19-related lung injury and inflammation will be explored in clinical trials elicited by Al Zoubi et al. (NCT04313322), Torres et al. (NCT04390139), and Monsel et al. (NCT04333368). Al Zoubi and colleagues will recruit 5 COVID-19 patients who will intravenously receive 3 doses of WJ-MSCs (1 × 10^6^ cells^/^kg). Each dose of WJ-MSCs will be given 3 days apart from the previous dose. Improvement of clinical symptoms in WJ-MSC-treated patients, including duration of fever, respiratory distress, pneumonia, cough, and sneezing, will be monitored for three weeks. This study is currently recruiting patients, and the estimated completion date is September 2020. Torres and colleagues will treat 30 SARS-CoV-2-infected patients with a combination of standard antiviral therapy and WJ-MSCs (1 × 10^6^ cells/kg), which will be intravenously injected at days 1 and 3. WJ-MSC-treated patients will be followed up on days 3, 5, 7, 14, 21, and 28. After the completion of the study, lung function of WJ-MSC-treated patients will be analyzed at 3 months, 6 months, and 12 months as long-term follow-up. Monsel and colleagues are currently recruiting 40 patients suffering from SARS-CoV-2-induced ARDS who are going to receive either WJ-MSCs (1 × 10^6^ cells/kg) or placebo (NaCl 0.9%) via a peripheral or central venous line at days 1, 3, and 5. Lung injury score, oxygenation index, and serum levels of immuno- and angiomodulatory cytokines (IL-1, IL-6, IL-8, TNF-*α*, IL-10, and TGF-*β*) will be determined to evaluate therapeutic effects of WJ-MSCs in attenuation of SARS-CoV-2-induced ARDS. The estimated study completion date is July 2021.

Ricordi and coworkers are currently recruiting patients with SARS-CoV-2-induced ARDS for participation in the clinical trial that will investigate safety and efficacy of UC-MSCs (NCT04355728). According to the study protocol, 24 patients will be randomly assigned to either the experimental or control group, respectively. Participants from the experimental group will be treated with two intravenous infusions of UC-MCSs (1 × 10^8^ cells/injection) in addition to standard of care treatment. The first infusion will be administered within 24 hours of study enrollment, and the second infusion will be administered within the next 48 hours. Patients from the control group will only receive standard of care treatment. Safety will be defined by the incidence of severe adverse events while efficacy will be determined by CT, biochemical, and laboratory parameters of lung, liver, and kidney function.

Aranzasti et al. (NCT04366271), McAuley et al. (NCT03042143), and Wang et al. (NCT04269525) are also going to evaluate the therapeutic effects of UC-MSCs in the treatment of COVID-19-related ARDS. The study led by Aranzasti and colleagues is currently recruiting 106 patients who will be randomly divided into the UC-MSC-treated and standard of care-treated groups. UC-MSC-treated patients will receive a single infusion of UC-MSCs. The exact number of injected UC-MSCs is not defined for this study. McAuley and coworkers will recruit 75 patients who will either receive UC-MSCs or placebo (Plasma-Lyte). Alteration in oxygenation index and incidence of serious adverse events will be monitored in UC-MSC-treated patients up to 28 days after UC-MSC infusion. The estimated study completion date is October 2022. In a clinical study led by Wang and colleagues (NCT04269525), SARS-CoV-2-infected patients will receive UC-MSCs (3.3 × 10^7^ cells/50 ml/bag, 3 bags each time). UC-MSCs will be infused intravenously at days 1, 3, 5, and 7. In addition to the assessment of pulmonary function, MSC-based immunosuppression will be determined by immunophenotyping of peripheral blood mononuclear cells and by the measurement of serum levels of TNF-*α*, IFN-*γ*, IL-2, IL-4, IL-6, IL-18, and IL-10. The estimated study completion date is September 2020.

## 6. Therapeutic Potential of MSC-Derived Extracellular Vesicles in Attenuation of SARS-CoV-2-Induced ARDS

Several lines of evidence demonstrated that most of the MSC-based beneficial effects in alleviation of lung injury were relied on immunomodulatory capacity of MSC-derived extracellular vesicles (EVs): apoptotic bodies, microvesicles, and exosomes (MSC-Exos) [18, 33]. Intravenously injected MSC-EVs accumulate in the lungs and, due to their nanosized dimension and lipid-enriched membrane, easily penetrate in the lung-infiltrate immune cells to modulate their phenotype and function [33]. MSC-EVs contain MSC-derived bioactive molecules (microRNAs (miRNAs), enzymes, cytokines, chemokines, and immunomodulatory and growth factors) that generate a tolerogenic phenotype in lung-infiltrated DCs and macrophages which results in enhanced expansion of immunosuppressive Tregs in injured lungs and leads to the alleviation of ongoing inflammation [18, 33]. As recently demonstrated by us and others [34, 35], in a similar manner as their parental cells, MSC-EVs efficiently attenuated detrimental immune responses in the lungs and promoted enhanced lung repair and regeneration. Reduced expression of costimulatory molecules, downregulated production of Th1- (IL-12, TNF-*α*, and IFN-*γ*) and Th17-inducing cytokines (IL-1, IL-6, and IL-23), and attenuated capacity of antigen-presentation capacity were noticed in DCs and macrophages isolated from the inflamed lungs of MSC-EV-treated animals [34, 35]. Consequently, significantly lower number of lung-infiltrating, inflammatory, TNF-*α*- and IFN-*γ*-producing Th1 cells and IL-17-producing Th17 cells and significantly higher number of immunosuppressive, IL-10- and TGF-*β*-secreting Tregs were found in MSC-EV-treated mice [34, 35].

In line with these preclinical data are results obtained in a clinical study conducted by Sengupta and colleagues [36]. Single intravenous injections of BM-MSC-derived Exos (15 ml, ExoFlo) were given to the 24 COVID-19 patients who met the criteria for moderate-to-severe ARDS. BM-MSC-Exos were well tolerated; no infusion reaction or adverse events were observed within the first 72 hours after BM-MSC-Exo administration [36]. Importantly, single intravenous infusion of BM-MSC-Exos significantly improved lung function, alleviated systemic inflammation, and increased total number of circulating neutrophils and lymphocytes in majority of COVID-19 patients [36]. BM-MSC-Exos efficiently attenuated SARS-CoV-2-related ARDS in 71% of BM-MSC-Exo-treated patients (17/24) who completely recovered within a week after intravenous administration of ExoFlo. Assessment of pulmonary function parameters revealed significantly improved oxygenation in BM-MSC-Exo-treated patients. An average pressure of arterial oxygen to fraction of inspired oxygen ratio (PaO2/FiO2) significantly increased in 20/24 of BM-MSC-Exo-treated patients, 3 days after ExoFlo infusion. The mean increase of PaO2/FiO2 from baseline was 191% at day 14 and was a strong predictor of hospital discharge for BM-MSC-treated patients. Biochemical analysis confirmed beneficial effects of BM-MSC-Exos. The mean reduction of CRP was 77%, the mean reduction of ferritin was 43%, and the mean reduction of D-dimer was 42% between baseline and values measured on day 5 [36]. Additionally, 5 days after systemic administration, BM-MSC-Exos significantly improved absolute number of circulating neutrophils and CD4+ and CD8+ T cells, as well as prevented development of severe immunodeficiency in SARS-CoV-2-infected patients. Despite these promising results, it should be noted that BM-MSC-Exo-based therapy was not effective in 29% (7/24) of COVID-19 patients. Four patients passed away for reasons unrelated to the BM-MSC-Exo administration, while 3 patients remained critically ill and required mechanical ventilation and intensive care [36].

In addition to the study published by Sengupta and colleagues, an additional clinical trial is going to investigate therapeutic potential of MSC-EVs in the treatment of COVID-19-related ARDS. Shahverdi and colleagues are currently recruiting 60 critically ill SARS-CoV-infected patients suffering from ARDS to participate in a randomized clinical trial which will evaluate efficacy of MSCs and MSC-EVs in the treatment of COVID-19-related ARDS (NCT04366063). Patients will be randomly assigned to 3 groups to receive the following: two doses of MSCs (1 × 10^8^ cells; intravenously at days 0 and 2; experimental group 1), two doses of MSCs (1 × 10^8^ cells) plus two doses of MSC-EVs (MSCs will be given at days 0 and 2 while MSC-EVs will be injected on day 4 and day 6; experimental group 2), and placebo (control group). Importantly, all patients from the experimental and control groups will receive conventional antiviral therapy and supportive care for ARDS treatment. Improvement of clinical symptoms, including duration of fever, respiratory distress, pneumonia, cough, sneezing, blood oxygen saturation, and 28-day mortality, will be analyzed to confirm efficacy of MSCs and their EVs in alleviation of SARS-CoV-2-induced ARDS during the 28-day follow-up.

## 7. Conclusions

Results obtained in two previously conducted clinical trials demonstrated that MSCs and their EVs efficiently suppressed detrimental immune responses in the lungs, alleviated ongoing inflammation, and significantly improved respiratory function in patients suffering from SARS-CoV-2-induced ARDS [32, 36]. Importantly, aggravation of COVID-19-related symptoms and adverse effects related to the infusion of MSC/MSC-Exos were not reported [32, 36], suggesting that administration of MSCs and their EVs was a safe and effective therapeutic approach for the treatment of SARS-CoV-2 infected patients.

Despite these promising results, it should be noted that the safety and efficacy of MSCs/MSC-EVs were, in both studies, evaluated in a small number of COVID-19 patients. Leng and colleagues evaluated therapeutic potential of MSCs in 7 COVID-19 patients, and almost all conclusions were made based on the analysis of MSC-based effects in one, critically ill patient [32]. Sengupta and coworkers examined the therapeutic potential of MSC-Exos in 24 patients and showed that MSC-Exos were not able to improve the respiratory function in 7 patients [36].

Additionally, it should be emphasized that MSCs are not constitutively immunosuppressive [37]. MSCs may alter their phenotype and become inflammatory cells if they engraft in the microenvironment with low levels of inflammatory cytokines, particularly IFN-*γ* and TNF-*α* [17, 37, 38]. These findings should be carefully considered in upcoming COVID-19-related clinical trials to avoid potential undesirable interactions between MSCs and antiviral drugs with immunomodulatory properties.

Since SARS-CoV-2 causes morbidity and mortality which escalate throughout the world, there might be temptation to progress with the clinical use of novel and unproven therapeutics. A large number of clinical trials are currently recruiting COVID-19 patients for MSC-related studies, and it is highly expected that these studies will provide important evidence about the therapeutic potential of MSCs in the treatment of SARS-CoV-2-induced ARDS. Therefore, with consideration to evidence-based science, MSCs and their secretome should be offered worldwide as a new remedy for the treatment of COVID-19 infection only if results from upcoming clinical trials confirm their safety and efficacy.

## Figures and Tables

**Table 1:** The list of the drugs which are tested as therapeutic agents for the treatment of COVID-19.

Therapeutic agent(s)	Mechanism of action	Expected therapeutic effect	Ref. no.
*Antiviral drugs*			
Remdesivir	Inhibition of the viral RNA-dependent RNA polymerase	Suppressed spreading of SARS-CoV-2	[7–14]
Sofosbuvir			
Ribavirin			
Mefuparib	Poly-ADP-ribose polymerase 1 inhibitor	Inhibition of SARS-CoV-2 replication	[11]
Toremifene	Inhibition of the SARS-CoV-2 spike glycoprotein	Inhibition of viral entry in AT2 cells	[11]
Camostat Nafamostat	Inhibitor of TMPRSS2	Suppressed spreading of SARS-CoV-2	[9, 10]
IFN-*β*-1b	Activation of interferon-stimulated genes in AT2 and immune cells	Suppressed replication and spreading of SARS-CoV-2	[7–10]
IVIG	Antibody-mediated neutralization of SARS-CoV-2	Inhibition of viral entry in AT2 cells	[11]
*Immunomodulatory drugs*			
Dexamethasone	Activation of histone deacetylase	Reduction of the harmful effects of cytokine storm	[7–11]
Sarilumab	Inhibition of IL-6:IL-6R signaling	Attenuation of IL-6-driven inflammation	[8–10]
Tocilizumab			
Anakinra	Inhibition of IL-1:IL-1R signaling	Attenuation of IL-1-driven inflammation	[12]
Baricitinib	Janus kinases inhibitor	Prevention of dysregulated cytokine production	[11]
Melatonine	Upregulation of superoxide dismutase; downregulation of nitric oxide synthase; suppression of NF-*κ*B expression	Inhibition of cytokine storm	[11]
*Combined therapy*			
Remdesivir+Dexamethasone			[10, 11]
IVIG+IFN-*β*+Dexamethasone			[11]
Remdesivir+Baricitinib			[10, 11]
IFN-*β*-1b+Ribavirin			[11–14]

Abbreviations: ACE2: angiotensin-converting enzyme 2; TMPRSS2: transmembrane protease serine 2; AT2: alveolar type II cells; IFN-*β*: interferon beta; IVIG: intravenous immunoglobulins; IL-6R: interleukin 6 receptor; IL-1: interleukin 1 receptor; NF-*κ*B: nuclear factor kappa-B.

## Data Availability

The data used to support the findings of this study are included within the article.
